# Associations between Religiosity, Spirituality, and Happiness among Adults Living with Neurological Illness

**DOI:** 10.3390/geriatrics3030035

**Published:** 2018-06-23

**Authors:** James B. Wade, Rashelle B. Hayes, James H. Wade, Jonathan W. Bekenstein, Kristin D. Williams, Jasmohan S. Bajaj

**Affiliations:** 1 Department of Psychiatry, Virginia Commonwealth University, Richmond, VA 23298, USA; rashelle.hayes@vcuhealth.org; 2 Virginia Tech Carilion School of Medicine & Research Institute, Roanoke, VA 24016, USA; jameshwade@vt.edu; 3 Department of Neurology, Virginia Commonwealth University, Richmond, VA 23298, USA; jonathan.bekenstein@vcuhealth.org (J.W.B.); kristin.williams@vcuhealth.org (K.D.W.); 4 Department of Internal Medicine, Virginia Commonwealth University, Richmond, VA 23298, USA; jasmohan.bajaj@vcuhealth.org

**Keywords:** psychological adjustment, happiness, spiritual well-being, neurological illness

## Abstract

The study examined the associations between religiosity, spirituality, and happiness in 354 outpatients suffering from neurological disorders. After accounting for severity of cognitive decline, physical activity level, depression severity, and demographic variables (i.e., subject age, sex, ethnicity, and marital status) multivariate linear regression revealed a unique association between the Spiritual Well-Being Existential Spirituality scale (SWBS ES), and not the SWBS Religious Scale (SWBS RS), with both the Pemberton Remembered Happiness Index (PHI R) (*p* < 0.001), and the Pemberton Experienced Happiness Index (PHI E) (*p* < 0.001). Interventions focused on existential spirituality may improve health related quality of life among adult medical patients with neurological illness.

## 1. Introduction

Fifty-one percent of Americans indicate that religion is a “very important” aspect of their lives [1]. Religious and spiritual dimensions that potentially enhance subjective well-being include beliefs about a beneficent God. There is a growing body of literature linking spirituality and better physical and mental health [2,3,4,5,6]. Studies [7,8,9] examining cancer patients’ psychological coping note that a spiritual belief system provides access to practical and emotional support during times of life stress. Spiritual beliefs appear to have a protective effect on depression in patients with heart failure [10], play a role in the emotional health of individuals recovering from stroke [11,12], and those with severe chronic disabilities [13]. Higher levels of religiosity and spirituality are associated with lower cardiovascular mortality [14] and enhanced health-related quality of life [15]. Possible mechanisms by which religiosity/spirituality impact health outcome include low rates of depression [16], better treatment compliance [17], improved immune function [18], and healthier diets [19]. Furthermore, religious involvement may provide a sense of attachment or belonging, and social identity, which mitigates role disruption and social isolation [20,21,22].

In the past it has been assumed that religiosity and spirituality were equivalent constructs [23,24]. Recent studies however have explored the differential impact of religiosity and spirituality on adjustment outcomes, such as happiness [25,26,27]. For example, in a study of cancer patients, Nelson [27] found spirituality to be more strongly associated with adjustment outcome (i.e., depression) compared to religiosity. Similarly, Purnell and Anderson [26] found in breast cancer survivors that spirituality was associated with health-related quality of life (HRQOL), but religiosity was not. In a study of HIV-positive patients Chang [28] found spirituality, but not religiosity, was associated with happiness. Interestingly, Nelson [29] found in a sample of HIV-infected adults that higher levels of religiosity were associated with greater intensity of depression, while the opposite was true for spirituality. Spirituality, in the Nelson study [29] was strongly and negatively associated with depression. In contrast with these findings, Kaufmann et al. [30] found higher levels of spirituality and private religious practices were associated with slower progression of Alzheimer’s disease. It remains to be determined whether the psychological adjustment of individuals with neurological illness is associated with religiosity, spirituality or both.

The goal of the present study was to examine the association between spirituality and religiosity with an index of psychological adjustment (e.g., happiness). In this study we used the Spiritual Well-Being Scale (SWBS) as measures of spirituality and religiosity [31,32]. Psychological adjustment was assessed using a contemporary measure of happiness, the Pemberton Happiness Index (PHI). Since levels of happiness may differ by age [33,34], depression severity [35,36,37,38], ethnicity [39], and marital status [40,41], we controlled for these demographic variables, along with current level of physical activity and severity of cognitive decline in individuals with neurological disorders. Our hypothesis was that higher levels of existential spirituality, rather than religiosity, would benefit individuals coping with major lifestyle threat, neurological illness, and experience greater levels of happiness.

## 2. Materials and Methods

The institutional review board at Virginia Commonwealth University approved the study protocol HM200112981.

### 2.1. Participants

Participants were patients at a southeastern United States university medical center outpatient psychological assessment center. Subjects were consecutive referrals for outpatient neuropsychological assessment by 2 of the authors (JB and KW). The project represents a sample of convenience. One of the authors (JBW) performed a retrospective chart review of patients seen by between 2015 and 2018. During this time period a total of 354 patients were evaluated and were on average 53 years of age (S.D. 16.14). As can be seen in Table 1 the subjects were predominantly Caucasian (72%), married (52%), women (62%). Subjects completed an average of 14 years of education. The most frequent neurologic diagnosis was hypoxia (35%).

### 2.2. Measures

The Spiritual Well-Being Scale (SWBS) is the most frequently used measure of spiritual well-being employed in clinical research today [42]. The SWBS yields three scores. The SWBS total score is a global measure of spirituality. This global score is comprised of 2 subscales. One is an assessment of one’s religious belief system, called the SWBS Religious scale (SWBS RS). The religious subscale clarifies the intensity of one’s religious conviction. For example an item from the scale is, “I believe God loves me and cares about me.” The other subscale is an assessment of one’s perception of spirituality in secular terms, called the SWBS Existential scale (SWBS ES). The existential subscale assesses one’s sense of connectedness to the world, and sense of life’s purpose. A sample item from the existential scale is, “I believe there is some real purpose for my life.” The SWBS RS items contain the word, “God.” The SWBS ES items do not include any religious language. Rather, items are worded in terms of spiritual transcendence. Half the items are reversed worded to eliminate response bias. The SWBS consists of 20 items, 10 of which represent the SWBS RS subscale; and 10 items on the SWBS ES subscale. Each item is scored from 1–6, with a higher number reflecting stronger spiritual conviction. As an index of internal consistency, coefficient alpha revealed high reliability. Across 7 samples, the internal consistency coefficients ranged from 0.82 to 0.94 (SWBS RS), 0.78 to 0.86 (SWBS ES), and 0.89 to 0.94 (SWBS) [43]. The SWBS shows good test-retest reliability. SWBS RS test-retest reliability coefficients across 4 studies, with 1–10 weeks between assessments, were 0.96, 0.99, 0.96, and 0.88. For SWBS ES the coefficients were 0.86, 0.98, 0.98, and 0.73. For the SWBS total score the coefficients were 0.93, 0.99, 0.99, and 0.82. The correlation coefficients between SWBS RS and SWBS ES subscale scores have ranged from about 0.20–0.71, indicating that the SWBS RS and SWBS ES subscales do not measure the same spiritual dimension [44]. This would be expected as it is possible for someone to perceive well-being both religiously and existentially at the same time, while not being identical. Factor analysis of the SWBS has consistently yield 2 factors [45,46]. All of the “God” items loaded on the SWBS RS factor; and all of the secular items loaded on the SWBS ES factor.

The Pemberton Happiness Index (PHI) is a contemporary integrative measure of psychological, social, and experienced well-being. Eleven items relate to different domains of remembered well-being (i.e., general, eudaimonic, hedonic, and social well-being) (PHI R). This scale reflects a subject’s enduring sense of life satisfaction. Therefore, this assessment is based on the subject’s memory and judgment of their lives. A sample item from this scale is, “I am satisfied with myself” Regarding the PHI R scale, a subject reads a sentence and then, using a Likert-type response (where zero is ‘totally disagree’ and 10 is ‘totally agree’), indicates the extent to which he/she agrees with each statement. PHI R scale is calculated by summing the first 11 items, which may vary from 0–10. The second PHI subscale, experienced well-being (PHI E) consists of 10 items related to the positive and negative events taking place the day before the assessment, and are scored dichotomously (response option: yes/no). Whereas the PHI R is an assessment of one’s general view of life, the PHI E is a contemporary assessment of the subject’s recent level of happiness. This scale assesses the emotional experience in “real-time,” rather than relying on memory to recall these states. A sample item from this scale is, “Yesterday something I did made me proud.” The 10 items from the PHI experienced well-being scale (PHI E) are converted into a single score ranging from zero to 10. In previous validation studies, Cronbach alpha (internal consistency) was above 0.89 [47]. Mean inter-item correlations of the PHI across 7 independent samples ranged from 0.31 (Turkey sample) to 0.56 (USA sample). With regard to convergent validity the PHI demonstrates a consistent pattern of correlations with other scales (i.e., SWLS = Satisfaction with Life Scale, SHS = Subjective Happiness Scale, PANAS = Positive and Negative Affect Schedules, SPWB = Ryff’s Scales of Psychological Well-Being, and the SWDL = Satisfaction With Domains of Life) assessing aspects of well-being [47]. The PHI has been validated in ten languages within their respective countries [47].

The Short Questionnaire to Assess Health-Enhancing Physical Activity (SQUASH) [48] developed by the Dutch National Institute of Public Health and the Environment, was also administered. This measure contains questions related to occupation, leisure time, household, transportation means, and other domains. Activities are given an intensity score (ranging from 1–9). The total number of minutes engaged in each activity is calculated by multiplying frequency (days/week) by duration (minutes/day). The higher the SQUASH score, the greater the level/duration of weekly physical activity. The reliability of the SQUASH in the Wendel-Vos study [48] was 0.58 (95% CI 0.36–0.74). De Hollander [49] used the Actiheart device (CamNTech, Cambridge, UK) to examine the validity of the SQUASH. The Actiheart measures daily life activities, calculates energy expenditure, and is able to make a distinction between the intensity of individuals’ activities [50,51]. These data support the SQUASH as a reliable and valid measure of physical activity level.

The Wechsler Abbreviated Scale of Intelligence–2nd Edition (WASI-II) [52] was administered to assess intellectual ability. Reliability coefficients for subtests’ internal consistency, as measured by a split-half method, ranged from 0.90–0.92. The average reliability coefficients were also excellent for the Full Scale Intelligence Quotient-4 (FSIQ-4), and FSIQ-2 composites, being 0.97 and 0.94, respectively. Test/retest reliability is good (0.83) to excellent (0.94) for the subtests, and excellent (0.90–0.96) for the composite scores. The WASI-II is intended to estimate intelligence close to a Wechsler Adult Intelligence Scale–4th edition (WAIS-IV) [53] FSIQ. WASI-II indices are correlated with scores on the original WASI [54], Wechsler Intelligence Scale for Children–4th edition (WISC-IV) [55], and the WAIS-IV [53] (0.71–0.92).

The Wechsler Test of Adult Reading (WTAR) [56] was used to estimate premorbid or characteristic intellectual ability. This instrument was developed and co-normed with the Wechsler Adult Intelligence Scale–3rd edition (WAIS-III) [57], and the Wechsler Memory Scale–3rd edition (WMS-III) [58]. Another advantage of the WTAR is its ability to estimate premorbid intelligence from demographic variables alone (i.e., age, highest educational level attained, gender, and ethnicity). Studies examining the validity of this estimate demonstrate improvement over that of the WTAR reading recognition subtest [56]. In the present study we subtracted a subject’s WASI-II Full Scale score from their demographic-based WTAR predicted score in estimating cognitive decline due to neurological illness.

Subjects also completed the Depression Visual Analog Scale (Depression VAS) [59,60,61], a 15-cm length scale with verbal anchor points, “none” and “the most severe imaginable.” Specifically, the patient was asked to, “place a mark along the scale reflecting the intensity of your depression.” The scale has been demonstrated to be reliable and valid [62]. Test retest reliability’s of the depression VAS have shown to be high, with values ranging from 0.70 to 0.90 [62]. Several studies support the reliability, validity, and sensitivity of the Depression VAS [63,64,65].

### 2.3. Data Analysis

To determine whether spiritual well-being is a factor in the happiness of individuals with neurologic illness, least squares multiple regression analysis was conducted using the statistical package for the social sciences (SPSS version 25; IBM [66]). There were 9 predictor variables in the regression model (i.e., age, sex, ethnicity, marital status, physical activity, cognitive decline, depression severity, and strength of spiritual and religious conviction). Two separate regression models were conducted. In the first model the Pemberton Remembered Happiness Index (PHI R) was the dependent variable; and in the second regression model the Pemberton Experienced Happiness Index (PHI E) was the dependent variable.

## 3. Results

Multiple regression was conducted to clarify the relationship between SWBS and both experienced (PHI E) and remembered (PHI R) happiness. In both sets of regression models, we statistically accounted for seven potential confounding variables such as severity of cognitive decline, level of physical activity, severity of depression, and demographic variables (age, ethnicity, marital status, and sex). PHI R (Shapiro-Wilk Test, *p* = 0.08), and PHI E (Shapiro-Wilk Test, *p* = 0.10) were determined to be normally distributed. Descriptive statistics for SWBS Total, SWBS Religious, SWBS Existential, PHI Remembered and PHI Experienced variables used in the regression analyses are shown in Table 2.

In the first regression (Table 3) model assessing the relationship between Remembered Happiness (PHI R) 7 independent variables were included, along with the SWBS Religious (SWBS RS) and Existential (SWBS ES) scales. This model examined whether there was a differential association between SWBS ES and SWBS RS with the PHI R.

A significant regression model was obtained (f (9,330) = 82.74, *p* < 0.001, with an R^2^ of 0.69). Therefore, in the overall model, independent variables accounted for 69% of the variance in PHI R. Review of the individual predictors, revealed age, ethnicity, sex, cognitive decline, physical activity, depression severity, and SWBS ES, to be uniquely associated PHI R. The SWBS religious subscale (RS) did not predict PHI R. SWBS ES was the strongest predictors of PHI R, uniquely accounting for 35% of the variance in PHI R. Therefore, SWBS ES uniquely accounted for 51% of the total variance in the regression model. For every one point increase in PHI R there is a 0.737 increase in the SWBS ES scale. Greater SWBS ES scores were associated with higher levels of happiness in neurologically ill patients. Ethnicity, cognitive decline, and physical activity uniquely accounted for less than 1% of the variance in PHI R. Similarly, subject age, sex, and depression severity each uniquely accounted for 1% of the variance in the model.

In the second regression model (Table 4) we included the same independent variables as the prior model and used the Pemberton Experienced Happiness Index (PHI E) as the dependent variable. This model clarified whether neurological patient’s PHI E differed as a function of their spiritual belief system. Consistent with the prior findings a significant regression model was obtained (f (9,330) = 21.52, *p* < 0.001, with an R^2^ of 0.35). Therefore, in the overall model independent variables accounted for 35% of the variance in PHI E. Review of individual predictors revealed age, ethnicity, depression severity, and SWBS ES, to be uniquely associated PHI E. Importantly, the SWBS religious subscale (SWBS RS) did not predict PHI E. Consistent with the prior findings SWBS ES was the strongest predictor of PHI E, uniquely accounting for 14% of the variance. Therefore, SWBS ES uniquely accounted for 40% of the total variance in the regression model. For every one point increase in PHI E there is a 0.462 increase in the SWBS ES scale. Greater SWBS ES scores were associated with higher levels of happiness in neurologically ill patients. Age and ethnicity uniquely accounted for approximately 1% of the variance in PHI E. Nevertheless, analysis of variance (ANOVA) revealed that the 3 ethnic groups (i.e., Caucasian, African American, and Asian) level of happiness (PHI E) did not differ (f (2,353) = 0.121, *p* = 0.886). In summary, regression analyses revealed secular, or existential spiritual beliefs (SWBS ES), to be strongly associated with both PHI R and PHI E.

## 4. Discussion

Despite the possibility that spirituality may play an important role in determining the psychological well-being of medically ill individuals, the authors are aware of no study examining its characteristics in patients with neurological disease. We found that subjects with a strong existential spiritual belief system enjoyed greater happiness while confronting the lifestyle threat of neurological illness. There was an inverse relationship between psychological adjustment, as measured by the Pemberton Happiness Index, and spirituality that persisted after taking into account a number of potentially confounding variables. While subject age, ethnicity, sex, and depression severity were associated with the Pemberton Remembered Happiness Index, their unique contribution was small. Similarly, with Pemberton Experienced Happiness Index as the dependent variable, subject age, sex, and depression severity each uniquely accounted for only limited variance. We accounted for functional status by assessing degree of cognitive decline and physical activity level in study subjects. Importantly, the relationship between spiritual beliefs and happiness was examined independently of neurological condition severity. Therefore, the higher levels of happiness experienced by individuals with strong spiritual beliefs was not a function of less neurobehavioral decline in study subjects. Our results are consistent with previous cross-sectional research showing a strong inverse relationship between spiritual well-being and psychological distress in patients with heart failure [10].

Importantly, the relationship between spiritual beliefs and happiness was related to secular, or existential spiritual beliefs. Specifically, the Spiritual Well-Being Religious Scale (SWBS RS) was not associated with either the Pemberton Remembered Happiness Index (PHI R) or the Pemberton Experienced Happiness Index (PHI E). For both the Pemberton PHI R and PHI E, the Spiritual Well-Being Existential Scale (SWBS ES) was the strongest predictor in the regression models. These data suggest spiritual beliefs, and in particular existential beliefs vs. religious conviction, are more strongly related to happiness in our population. The strength of an individual’s existential spiritual belief system may impact coping with lifestyle adversity. Our findings are consistent with previous research that emphasized the role of spirituality, compared to religiosity, in predicting psychological adjustment in medical patients [29]. For example, Chang [28] and Galea [25] found spirituality, not religiosity, to be associated with psychological adjustment. One possible explanation for the nonsignificant relationship between religiosity and happiness, is that religious spirituality relies upon an external superior force, such as a beneficent God, to account for well-being. In contrast, existential spiritual beliefs are a measure of an individual’s personal or private strength in coping with lifestyle adversity. The existential spirituality measure may better reflect individual self-efficacy, and therefore may have a stronger association with both remembered and experienced levels of happiness. With regard to the two Pemberton Happiness Indices, a stronger association was noted between the Spiritual Well-Being Existential Scale and the Pemberton Remembered Happiness Index. The Pemberton Experienced Happiness Index is determined upon review of events taking place solely within the previous 24 h. The weaker association between the Pemberton Experienced Happiness Index and existential spirituality may reflect the fact that neurological disease-related burden limited study subject’s participation in activities, over the previous 24 h, associated with happiness. In contrast, with the Pemberton Experienced Happiness Index, the Pemberton Remembered Happiness Index involves a several year reflection regarding life-satisfaction.

The global population is getting older and living to advanced ages. For the first time, most people in the world can expect to live into their sixties [67]. Given increasing longevity, ways in which good health in old age can be promoted is important. Although research suggests religiosity/spirituality is a determinant of longevity, it remains to be seen whether they contribute to healthy life expectancy. The largest body of literature concerning the impact of religiosity/spirituality on health focuses on coping with stressful life events. This is important for older persons, because they are confronted by the loss of a loved one, decline in mental and physical function, and ultimately their own mortality. By enhancing life meaning, spirituality has been shown to bolster life satisfaction, optimism and self-esteem, which serves to reduce the stress of life events [68,69,70]. Using techniques that enhance an individual’s existential spiritual belief system, that are simple to implement, and require only a few minutes of daily practice, has demonstrated success in improving the health related quality of life (HRQOL) of medical patients, and practice associated changes in functional brain activity may prove efficacious. The Loving Kindness Meditation promotes acceptance and compassion for all living things [71]. The practitioner cultivates openness, and selfless love towards themselves and others [72]. This specific meditation technique is associated with enhanced levels of happiness, diminished depression and social anxiety [73]. For example, Bajaj et al. [74] evaluated the efficacy of the Loving Kindness Meditation in patients suffering from end-stage liver disease, along with their caregivers. After 4 meditation sessions depression was reduced in study participants, along with reduced burden and improved sleep hygiene for their caregivers. Functional magnetic resonance imaging (fMRI) [75] with Loving Kindness Meditation practitioners resulted in enhanced functional connectivity involving the cingulate cortex/precuneus, and left inferior frontal gyrus regions. Lutz, Brefczynski-Lewis, Johnstone, and Davidson [76] using fMRI found increased activation in similar brain regions (i.e., cingulate cortex and insula). Taken together these data suggest the Loving Kindness Meditation may impact affective processing by altering neural network connectivity. Future research is needed to clarify whether this meditative technique can enhance existential spirituality.

Limitations of the present study need to be considered when interpreting the results. The first concerns how we chose to assess spirituality. The SWBS is a measure of the cognitive/affective dimension of spirituality. We did not measure behavioral manifestations of spirituality, such as how often someone attends church, or the frequency/duration of daily prayer. We limited our assessment to one component of spirituality, the affective/cognitive domain. In addition, there may be an overlap between the SWBS and measures of psychological well-being, such as the PHI. The relationship between psychological health and spirituality has been previously reported in various medical populations using different of measures to assess emotional suffering [77,78]. Importantly, in our analyses the association between spirituality and happiness was evident after we accounted for severity of depression, suggesting that spiritual well-being can uniquely contribute to overall psychological function. Also, the unique relationship between existential, and not religious beliefs, and happiness, speaks to the discriminative nature of this of this association.

The fact that this study is cross-sectional nature limits our ability to draw conclusions about the direction of the association. We cannot state based on the current data that existential spirituality causes changes in psychological adjustment, or that better coping strategies lead to a strengthening of existential spirituality. These findings need to be replicated in a larger population, using a longitudinal design to clarify whether this potentially protective effect of spirituality on psychological adjustment can be maintained over time. Also some form of selection bias may have taken place. All subjects were referred by 2 of the authors (neurologists) for outpatient neuropsychological assessment. It would be important to examine the generalizability of the present findings with other medical groups, and not to rely on a sample of convenience. As the sample consisted of largely married females our findings may not generalize to other more diverse populations. The study focused on affect/cognitive-based indices of spirituality. To improve our understanding of the relationship between religiosity and happiness future studies should include behavioral measures of religiosity, such as frequency of attending religious services, and amount of time spent in prayer.

In conclusion, existential spiritual beliefs were associated with happiness, even after controlling for severity of depression, cognitive decline, physical activity level, and demographic variables (i.e., age, sex, marital status, and ethnicity) in a group of outpatients suffering from neurological illness. Given the demonstrated unique relationship between spiritual well-being to the overall psychological health, and quality of life of patients with neurological illness, the spiritual existential dimension of living with neurological illness should receive more attention in the care of these patients. Interventions focused on existential well-being may be of benefit to improve health of adult medical patients with neurological illness.

## Figures and Tables

**Table 1:** Demographic and Neurological Characteristics of the Participants.

Variable	Demographic Data
Age, mean ± S.D., range	53.20 (16.14), 37–85
Sex, M = men, F = female (%)	134 M (37) 220 F (62)
Ethnicity, C = Caucasian, AA = African American, A = Asian American *n* (%)	257 C (72) 86 AA (24) 11 A (3)
Marital Status, S = single, M = married, D = divorced, W = widow *n* (%)	79 S (22), 185 M (52) 62 D (17), 28 W (7)
Neurological Illness *n* (%)	
Seizures	16 (4.5)
Cerebral Vascular Disease	30 (8.4)
Alzheimer’s Type dementia	12 (3.4)
Multiple Sclerosis	16 (4.5)
Traumatic Brain Injury	40 (11.2)
Hypoxia	126 (35.4)
Metabolic Disease	27 (7.6)
Tumors	13 (3.7)
Frontal Dementia	7 (2.0)
Hydrocephalus	2 (.6)
Other	44 (12.0)
HIV Dementia	4 (1.1)
Pain	13 (3.7)
Parkinson’s Disease	4 (1.1)

**Table 2:** Summary of Spiritual Well-Being (SWBS) and Pemberton Happiness Index (PHI) Scales.

Scale Name	Min. to Max	Mean	SD
Spiritual Well-Being Scale (SWBS)			
Total Score	20–120	68.39	20.51
Religious Scale	10–60	44.55	14.59
Existential Scale	10–60	43.74	10.23
Pemberton Happiness Index (PHI)			
Remembered Scale	0–110	56.90	23.46
Experienced Scale	0–10	5.24	2.37

**Table 3:** Summary of Regression Analysis for Variables Predicting Pemberton Remembered Happiness Index (PHI R).

Variable	B	SE B	β	R^2^
Age	0.122	0.046	0.084	0.006 **
Ethnicity	3.166	1.459	0.068	0.004 *
Marital Status	1.469	0.762	0.062	0.003
Sex	−4.500	1.530	−0.093	−0.008 **
Cognitive Decline	0.166	0.066	0.079	0.005 **
Physical Activity	0.002	0.001	0.068	0.004 *
Depression	−0.551	0.142	−0.131	−0.013 ***
SWBS Religious Scale	−0.052	0.055	−0.032	−0.001
SWBS Existential Scale	1.682	0.086	0.737	0.345 ***

Note: * *p* < 0.05, ** *p* < 0.01, *** *p* < 0.001.

**Table 4:** Summary of Regression Analysis for Variables Predicting Pemberton Experienced Happiness Index (PHI E).

Variable	B	SE B	β	R^2^
Age	0.018	0.007	0.120	0.013 **
Ethnicity	0.415	0.210	0.089	0.007 *
Marital Status	0.149	0.110	0.062	0.003
Sex	−0.669	0.220	−0.137	0.017 **
Cognitive Decline	−0.016	0.009	−0.074	0.005 **
Physical Activity	−5.53	0.000	−0.022	−0.001
Depression	−0.072	0.020	−0.170	−0.023 **
SWBS Religious Scale	0.005	0.008	0.034	0.001
SWBS Existential Scale	0.106	0.012	0.462	0.138 ***

Note: * *p* < 0.05, ** *p* < 0.01, *** *p* < 0.001.
